# Building Trust and Resilience: Bhutan's Approach to Risk Communication During the COVID‐19 Pandemic

**DOI:** 10.1002/puh2.70039

**Published:** 2025-03-12

**Authors:** Ugyen Tshering, Tandin Dendup, Sonam Wangda, Sonam Wangdi

**Affiliations:** ^1^ Department of Public Health Ministry of Health Thimphu Bhutan; ^2^ Planning and Policy Division Ministry of Health Thimphu Bhutan; ^3^ Department of Health Services Ministry of Health Thimphu Bhutan; ^4^ WHO European Centre for Preparedness for Humanitarian and Health Emergencies Istanbul Turkey

**Keywords:** Bhutan, COVID‐19, pandemic preparedness, risk communication, system‐level reforms

## Abstract

Throughout the COVID‐19 pandemic, effective risk communication emerged as pivotal in fostering positive behavioral changes that aligned with the evolving evidence and stages of the pandemic. It stood alongside key strategies like enhanced surveillance, extensive testing, stringent quarantine, and strategic case management in Bhutan's response. Over the last 3 years of the pandemic, we have gained profound insights into risk communication's impact. This article aims to illuminate diverse approaches in managing public information during the pandemic. The authors also suggest potential research agendas and policy and system‐level reforms in the realm of risk communication.

## 1 Background

Bhutan, a nation vulnerable to a range of biological and natural hazards, has recognized the imperative of enhancing its emergency preparedness and response systems. As an essential component of its preparedness strategy, the Ministry of Health (MoH) has directed its attention toward equipping health professionals with the skills necessary for effective communication during health emergencies. In 2016, a dedicated section on risk communication was integrated into the Health Emergency and Disaster Contingency Plan (HEDCP) [1].

MoH initiated its first‑ever training on risk communication for health managers in 2017 [2]. This training has since become an annual event. Notably, the Risk Communication Guideline for the Health Sector was published in early 2019 [3], a year before the emergence of the COVID‐19 pandemic. Furthermore, MoH has been providing training to journalists since 2018 in the fields of data interpretation, scientific communications, and ethical reporting on public health topics. Such proactive approaches proved invaluable when Bhutan confronted the unprecedented challenges posed by the COVID‐19.

Nevertheless, when Bhutan grappled with the COVID‐19 challenges, the novel nature of the virus brought about considerable uncertainty. During the initial stages of the pandemic, its behaviors and impacts remained largely uncertain. The combination of uncertainty, a constrained national capacity to handle the infodemic, and widespread smartphone usage in the country contributed to the dissemination of misinformation, assumptions, and myths. These factors presented substantial challenges to the nation's endeavors to curb the pandemic.

In this article, we explore Bhutan's experience with risk communication during its COVID‐19 response. We also suggest potential research agendas and policy and system‐level reforms to enhance future preparedness for health emergencies or disasters.

## 2 Approaches and Insights During the Pandemic

### 2.1 Leadership and Stewardship

During the pandemic, the Health Minister served as the Incident Commander for the Health Emergency Management Committee as per the HEDCP. Various teams, including the National COVID‑19 Media and Risk Communication Team (NCMRT), were established in early February 2020. These teams provided essential risk communication stewardship throughout the COVID‐19 outbreak. To stay abreast with the evolving global disease landscape, team members proactively participated in WHO's webinars and courses.

Besides creating strategies to tackle misinformation and myths, these teams were tasked with monitoring global scientific literature for pandemic updates. NCMRT's task was to adapt and deliver this information effectively to the Bhutanese population in a timely manner. Surveillance, testing, quarantine, vaccination, morbidity, and mortality data were collected and shared via MoH's social medias. Furthermore, MoH collaborated with influential figures to enhance the reach of risk communication efforts [4].

The NCMRT organized regular press briefings to keep the public informed about the evolving COVID‐19 situation. The first press briefing on January 29, 2020 outlined MoH's major preparedness interventions to prevent the virus entry into the country in line with the IHR 2005. The updates were especially frequent during the peak of the pandemic in mid‐2020, with daily press briefings [5]. The proactive communication strategy raised awareness about COVID‐19 and promoted adherence to crucial public health and social measures.

### 2.2 Leveraging Social Media and Beyond

The extensive usage of smartphones and internet services throughout the country [6] has not only provided unparalleled access to information but also exposed individuals to a flood of misinformation about COVID‐19. Nevertheless, MoH turned this smartphone accessibility to its advantage by actively utilizing its social media presence on diverse platforms. Primarily leveraging on common platforms, like Facebook, WhatsApp, Instagram, and WeChat, the team not only distributes timely and crucial information but also counteracts false information. The use of these platforms was promising for effectively disseminating public health information and fostering positive behavioral changes, as reported in a previous study [7].

From early 2020 to early 2022, MoH's Facebook page saw substantial growth in followers, increasing from 15,500 to over 136,000. Meanwhile, MoH's Instagram account gained 20,800 followers [4]. MoH also actively shared materials through both official and informal groups on WhatsApp and WeChat. In terms of social media reach, these handles collectively reached 8.8 million people since February 2020, resulting in 11.4 million engagements [4].

**FIGURE 1:**
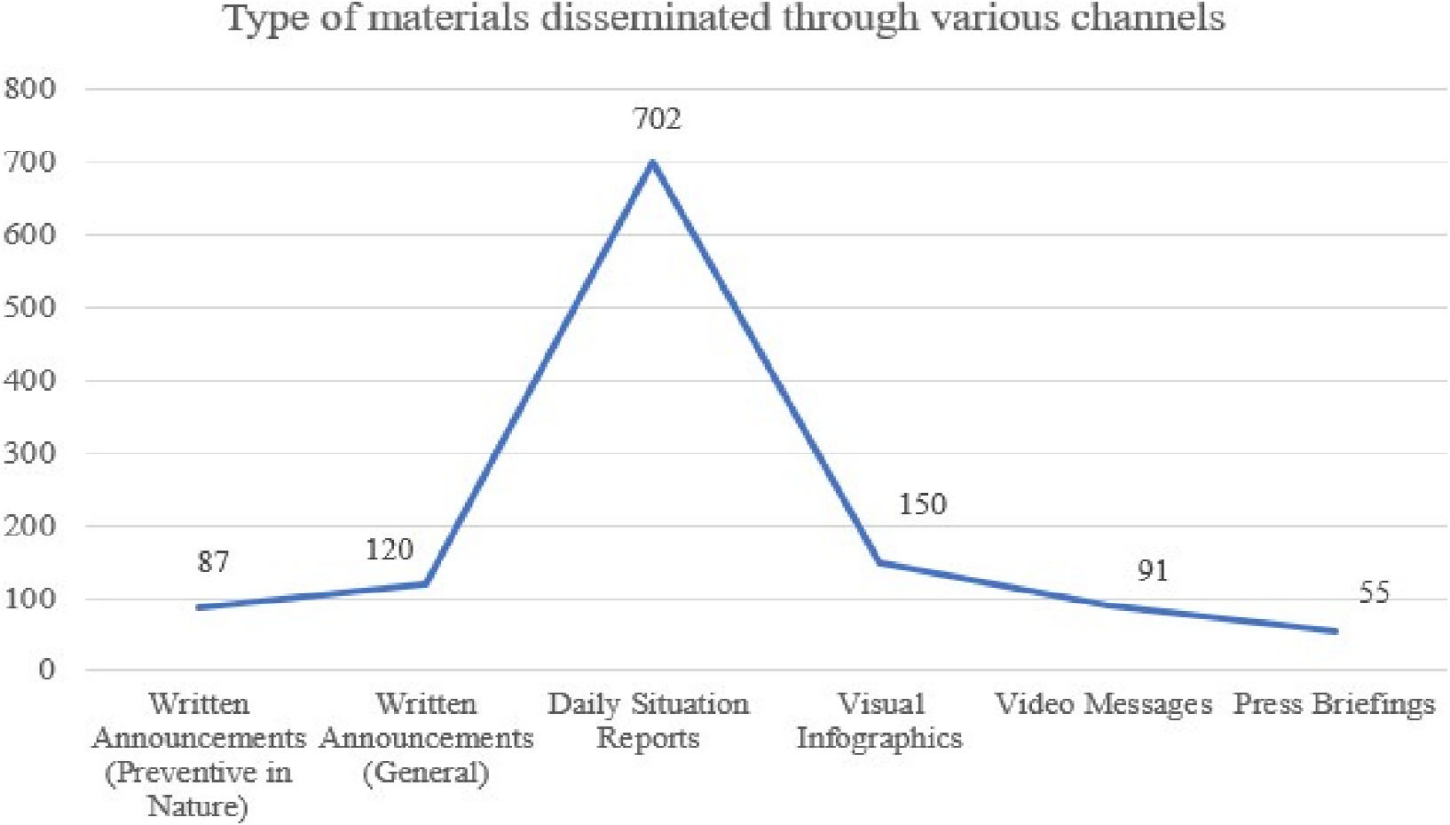
Types of materials disseminated through various channels [4].

To reach a wider audience, messages advocating public health measures were aired across various platforms, including radio channels, ensuring accessibility for grassroots communities. Many video messages were also translated into major local dialects. Various hotlines set up by the government were used to collate people's doubts and queries. Figure 1 provides a broad overview of the different types of materials that were produced and disseminated.

### 2.3 Community Engagement

One of the early strategic approaches for COVID‑19 responses adopted was to initiate a whole of government/society approach to combat the virus [1]. The approach placed significant importance on community engagement as a key element in achieving an effective response to the pandemic. Considering its importance, significant efforts were made to engage critical stakeholders and target populations in Bhutan's response to the pandemic.

Key populations that would be impacted by the pandemic were identified and broadly classified them into affected parties, interested parties, and vulnerable groups highlighting the associated risks, impacts, and desired interventions for each category [8]. Communities were prioritized at the core of all public health interventions throughout the pandemic. Community engagement efforts were intensified by engaging local government leaders, associations of elderly and disabled people, business communities, journalist associations, civil society organizations, religious figures, and social media influencers.

With the primary objective of fostering national unity during and after the COVID‐19 era, MoH initiated the “Our Gyenkhu (Our Responsibility)” campaign. Under this campaign, various social media influencers such as popular actors, bloggers, artists, and sports personalities were engaged to combat misinformation and promote awareness about COVID‐19. This initiative was not only strategically designed to reinforce the COVID‐19 mitigation efforts but also initiated a new nation‐building movement through a collaborative approach of “whole of nation and society approach.” This initiative significantly strengthened risk communication during the peak of the national response to the pandemic. It also fostered a sense of responsibility among Bhutanese citizens, encouraging active participation in nation‐building efforts that extend beyond the post‐COVID‐19 era.

## 3 Suggestions for Research Agenda

A potential research agenda could focus on studying the impact of the established communication channel, how it enhances collaboration between the COVID‐19 technical team and other response units, and its effect on public awareness and behavior during health crises. Such research aims to inform policy decisions, improve communication strategies, and strengthen overall future response capabilities.

Leveraging existing MoH's social media platforms proved to be a game changer in reaching out to the masses in a timely manner. However, the availability of various social media platforms also served as a vehicle of misinformation, resulting in confusion and panic to both frontline workers and the general public. Therefore, there is a need to dip down on the studies on how to streamline the availability of various social media platforms for sharing accurate and timely information.

When the COVID‐19 pandemic ended, the NCMRT team was disassembled. It is crucial not only to document the innovations and experiences gained but also to implement and integrate these learnings into the system. This step not only ensures sustainability but also builds resilience for future emergencies. Hence, there is a need to study how effectively the systems have adopted the lessons learned and establish methods to transfer the acquired knowledge and skills to the health communication team handling routine and risk communication during smaller outbreaks.

## 4 Suggestions for Policy and System‐Level Reforms

The COVID‐19 pandemic served as a true test of Bhutan's preparedness and capability to respond to health emergencies. It offered valuable insights into the need for health systems to swiftly adapt to effectively combat global health emergencies. In light of the initial challenges faced during the pandemic, there is a critical need for policy reforms in risk communication within Bhutan's health emergency response framework. Priority areas for policy focus could include enhancing capacity building in risk communication and infodemic management, including creating essential audiovisual content and infographics.

COVID‐19 frontline workers were educated about the significance of disseminating accurate information and remaining vigilant against false reports and news. Social media creatives on COVID‐19 were shared with their social media groups so that everyone from the health sector communicated in a uniform voice reinforcing the principle of a single source of truth for communication. At times, the influx of information from diverse sources confused them about its validity. Nevertheless, they played a pivotal role in bridging the communication gap between the general public and health authorities.

The above approaches underscore the importance of implementing robust policy reforms in communication strategies. Strengthening these strategies requires the establishment of a unified communication plan, offering comprehensive guidelines for information sourcing, verification protocols, and consistent messaging across all personnel involved. Alongside this, there is also a need to create a centralized information hub. This hub will provide a reliable and easily accessible repository of verified information for all stakeholders, thereby mitigating the confusion caused by diverse information sources.

During the press briefings, sign language interpretation was also integrated through national television. Such practices have to be institutionalized during national emergency broadcasts, ensuring inclusivity and accessibility for the deaf community during critical situations like pandemics. By mandating this provision, it establishes a more equitable approach to information dissemination, catering to diverse communication needs during emergencies.

Active engagement with diverse communities proved pivotal in our effective response to COVID‐19, particularly in the domains of risk communication and infodemic management. The MoH's strategy successfully disseminated crucial COVID‐19 messages to grassroots levels, prompting positive behavioral change. Leveraging these established community engagement systems in future emergencies is imperative; it fosters synchronization, minimizes conflicting information, and presents a unified front in our risk communication endeavors.

## Author Contributions

Ugyen Tshering conceptualized and designed the study, conducted literature search, and drafted the manuscript. Tandin Dendup conducted literature search and revised the manuscript. Both Sonam Wangda and Sonam Wangdi revised the manuscript.

## Ethics Statement

The authors have nothing to report.

## Consent

All co‐authors approved the publications of this article.

## Conflicts of Interest

The authors declare no conflicts of interest.

## Data Availability

The authors have nothing to report.
